# Live imaging of *Drosophila melanogaster* neural stem cells with photo-ablated centrosomes

**DOI:** 10.1016/j.xpro.2022.101493

**Published:** 2022-06-23

**Authors:** Alexandre Thomas, Régis Giet

**Affiliations:** 1 Univ Rennes, CNRS, INSERM, IGDR (Institut de Génétique et Développement de Rennes)-UMR6290, ERL U1305, 35000 Rennes, France

**Keywords:** Cell Biology, Developmental biology, Microscopy, Model Organisms, Neuroscience, Stem Cells

## Abstract

*Drosophila* neural stem cells (NSCs) divide asymmetrically to generate siblings of different sizes. This model system has proved helpful in deciphering the contribution of polarity cues and the mitotic spindle in asymmetric cell division. Here, we describe a technique we developed to flatten cultured *Drosophila* brain explants to accurately image the cytoskeleton in live NCSs. We also describe our approach to efficiently remove centrosomes by laser photo-ablation and to measure daughter cell size after NSC division.

For complete details on the use and execution of this protocol, please refer to Thomas et al. (2021).

## Highlights

• Preparation of incubation chambers for tissue imaging
• Preparation of live *Drosophila melanogaster* larval brain explants
• Laser photo-ablation of centrosomes in NSCs
• Imaging and analysis of NSCs devoid of their apical or basal centrosome

## Abstract (editor highlights)

Publisher’s note: Undertaking any experimental protocol requires adherence to local institutional guidelines for laboratory safety and ethics.

## Graphical abstract

**Figure:**
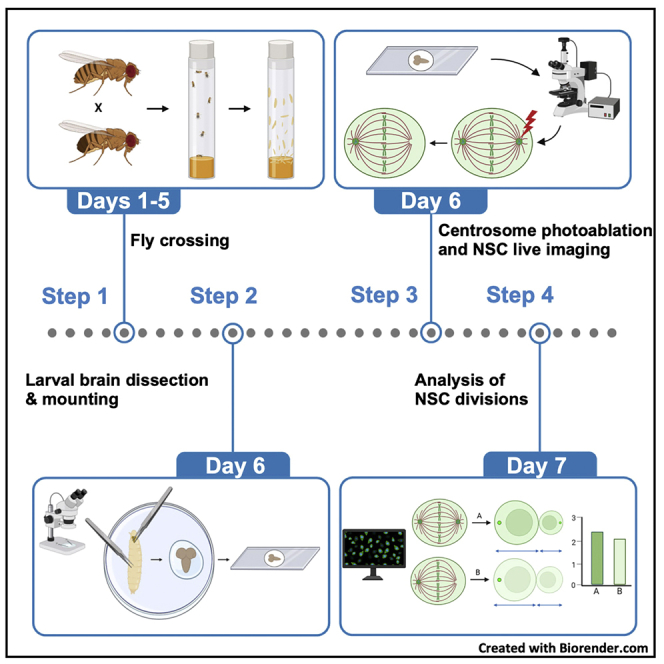

## Before you begin

Asymmetric cell division (ACD) is an essential mechanism that generates cell diversity, drives tissue repair and therefore maintains tissue homeostasis (Knoblich, 2010). For example, many cell types, including stem cells, are characterized by the asymmetric distribution of specific mRNAs or proteins between daughter cells. This biased delivery is essential for specific fate acquisition (Homem and Knoblich, 2012; Gallaud et al., 2017). *Drosophila* larval neural stem cells (NSCs) (also known as neuroblasts, NBs) display an asymmetric positioning of the division furrow. Their division generates a large self-renewing NSC and a smaller ganglion mother cell (GMC) committed to differentiation (Thomas et al., 2021; Connell et al., 2011; Albertson and Doe, 2003). This cell size difference, together with the available transgenic lines, mutants, and numerous tools available in *Drosophila melanogaster,* make NSCs a valuable model to study the molecular mechanisms of asymmetric cell division. Each *Drosophila* larval brain explant can be kept alive for several hours for imaging experiments and contain an average of 100 NSCs that exhibit rapid cell cycles (45–60 min). Here we describe a protocol to perform centrosome laser-ablation of mitotic NCSs and to investigate daughter cell size asymmetry following cytokinesis. This protocol could be adapted to monitor cytoskeleton dynamics in other tissues.

### Preparing flies

**Timing: 5 days**

The first step is to produce larvae that simultaneously express several markers: a plasma membrane marker, intended to measure cell diameters before and after division (PH-PLCδ-GFP), a centrosome marker that can be easily targeted by the laser during the photo-ablation step (GFP-Aurora A), and a microtubule marker to confirm the absence of astral microtubule nucleation at the mitotic spindle poles (RFP-tubulin).

1. Cross 10 virgin females carrying homozygous copies of the Ubi-RFP-tubulin transgene (genotype; *w*^*1118*^; Ubi-RFP-Tub) with 3–5 males carrying homozygous copies of the GFP-AurA;H2A-GFP;Ubi-PH-PLCδ-GFP transgenes (*w*^*1118*^;pAur-GFP-AurA,H2A-GFP;Ubi-PH-PLCδ-GFP) in a fresh vial supplemented with fresh yeast paste.
2. Transfer the adults every 2–3 days into new vials supplemented with fresh yeast paste.

### Preparation of the incubation chamber

**Timing: 15–20 min**

**Figure 1:**
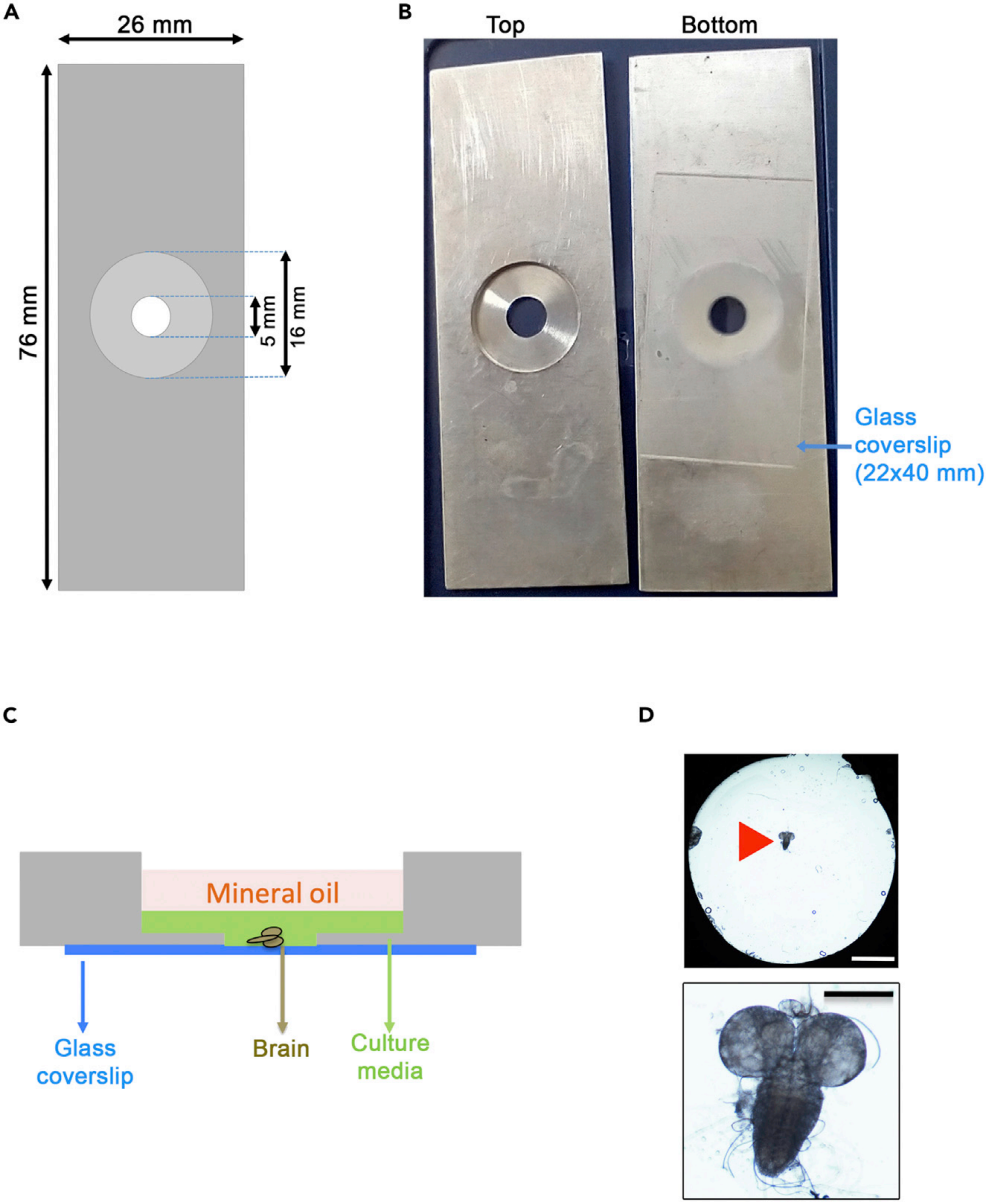
Preparation of incubation chambers for NSC centrosome ablation and live imaging (A) Scheme of the stainless-steel slide used for incubation chambers: Length: 76 mm, Width: 26 mm, Height: 1 mm. The central part is composed of a large 16 mm diameter well (0.8 mm high) inside which a 5 mm well (0.2 mm high) is centered. (B) Photograph of an incubation chamber seen from above (left) and below (right). The bottom view shows the incubation chamber on which a 22 × 40 mm coverslip has been fixed with VALAP. (C) Side view of the incubation chamber with a brain sample before laser ablation. After dissection, the larval brain is deposited with culture medium in the central well using a pipette. The sample is covered with mineral oil to prevent evaporation. Excess culture medium can be aspirated at this point to slightly flatten the brain. (D) Top view of the central well with a live dissected brain (red triangle) in the 5 mm incubation chamber by phase contrast microscopy (upper panel, scale bar: 1.6 mm). An enlargement view of the brain is also shown in the lower panel and shows the two optic lobes and the ventral ganglion. Scale bar: 200 μm.

***Note:*** Coverslips are stored in 95% ethanol. The melted VALAP must not settle inside the well nor spill on the outside. If this happens, too much VALAP had been used and should be wiped off immediately and the procedure repeated with a new glass coverslip using less VALAP. Incubation chambers should be prepared in advance and store away from dust.

***Note:*** We strongly recommend using one incubation chamber per imaging experiment. The stainless-steel slide can be reused indefinitely. After the imaging, wipe off the mounting medium and heat the slide upside down on the heating block at 70°C. Carefully remove the coverslip and immediately wipe the VALAP off the steel slide to prepare a new incubation chamber.

3. The incubation chamber is composed of a stainless-steel slide on which a 40 × 22 mm coverslip has been glued with VALAP (1/3 Vaseline, 1/3 Lanolin, 1/3 Paraffin) (Figures 1A and 1B). The gluing of the coverslips is done as following.
  a. Place the slides upside down on a dry bath incubator at 70°C.
  b. Apply a small amount of VALAP near the central well with a spatula.
  c. Place the coverslip on the melted VALAP and let the VALAP diffuse by capillary action.
  d. Return the incubation chamber at 18°C–22°C to harden the VALAP and to maintain the chamber with the coverslip (Figure 1B).

## Key resources table

**Table:** 

REAGENT or RESOURCE	SOURCE	IDENTIFIER
**Antibodies**		

Rabbit polyclonal anti-Cnn (dilution 1/1000)	Tim Megraw Lab (Zhang and Megraw, 2007)	PMID: 17671162
Rat anti-α-tubulin clone YL1/2 (dilution 1/1000)	Millipore	RRID: AB_2617116
Rabbit polyclonal anti-phospho Histone H3 (ser10) (dilution 1/1000)	Millipore	RRID: AB_565299

**Chemicals, peptides, and recombinant proteins**		

Lanolin	Thermo Fisher Scientific	Ref. A16902
Paraffin	Merck	Ref. 1.07300
Vaseline	Sigma-Aldrich	Ref. 16415
Mineral oil	Sigma-Aldrich	Ref. M8410
Schneider’s *Drosophila* medium	Thermo Fisher Scientific	Ref. 21720-024
FCS	Eurobio	Ref. CVFSVF00-01
Penicillin/Streptamycin	Gibco	15140-122
PBS	EUROMEDEX	Ref.ET330
Formaldehyde	Sigma-Aldrich	Ref. F1635
Triton X-100	Sigma-Aldrich	Ref. X100

**Experimental models: Organisms/strains**		

*w; pAur*-GFP-AurA, H2A-GFP*;* Ubi-PH-PLCδ-GFP (adult males)	Regis Giet Lab, (Caous et al., 2015) Antoine Guichet Lab; (Claret et al., 2014)	PMID: 26568519 PMID: 24768049
*w* ; Ubi-RFP-α-tubulin;+ (virgin females)	Renata Basto Lab; (Basto et al., 2008)	PMID: 18555779
*w; pAur*-GFP-AurA, H2A-GFP*/* Ubi-RFP-α-tubulin*;* Ubi-PH-PLCδ-GFP/+ (third instar larvae)		

**Software and algorithms**		

Fiji	fiji.sc	https://imagej.net/software/fiji/
Prism version 7	GraphPad	https://www.graphpad.com/scientific-software/prism/

**Other**		

Stainless steel slides	GREM (https://www.grem-paris.fr)	NA
LEICA SP5 confocal microscope	Leica	N/A
Nikon SMZ745 stereomicroscope	Nikon	N/A
Fine tweezers	Oxford Instruments	Ref. AGT5130

## Materials and equipment

**Timing: 15 min for the fresh fixative solution**

**Timing: 5 min for the PBST**

**Timing: 60 min for the culture medium**

Preparation of reagents.

**Table:** Fixative solution

Reagent	Final concentration	Amount
Tris PH 6.8 (1 M)	10 mM	0.1 mL
KCl (1.87 M)	183 mM	1 mL
EDTA (1 M)	1 mM	0.01 mL
Formaldehyde 37%	10%	3 mL
Triton X-100 100%	0.01%	1 μL
ddH_2_O	n/a	5.89 mL
**Total**	**n/a**	**10 mL**

***Note:*** Prepare a fresh solution at 20°C–25°C for each day of the experiment.

**Table:** PBST

Reagent	Final concentration	Amount
PBS	n/a	49.95 mL
Triton X-100	0.1%	0.05 mL
**Total**	**n/a**	**50 mL**

***Note:*** Store at 4°C for a maximum of 48 h.

**Table:** Culture medium

Reagent	Final concentration	Amount
Schneider medium	n/a	449.5 mL
Fetal Calf Serum	10%	50 mL
Penicillin/Streptomycin 100000 UI/mL	100 UI/mL	0.5 mL
**Total**	**n/a**	**500 mL**

***Note:*** Store in 1 or 2 mL aliquots at −20°C. Use a fresh aliquot for each day of experiment. Centrifuge the culture medium at 10,000 × *g* for 1 min to remove debris and use the supernatant.

## Step-by-step method details

### Collecting larvae expressing fluorescent proteins

**Timing: 15 min**

1. Larval collection.
  a. Start collecting wandering third instar larvae with fine forceps 96–120 h after egg laying.
  b. Leave each larva in 0.1 mL drops of PBS in a Petri dish for subsequent brain dissection.

***Note:*** Note that all larvae express one copy of the GFP-AurA, H2A-GFP, PH-PLCδ-GFP and α-tubulin-RFP proteins. We recommend using young wandering third instar larvae. Brain lobes contain a defined number of NSCs (≈100). These 100 cells are spatially much closer to each other in younger brains compared to older brain that have generated much more GMCs. Therefore, young wandering third instar larvae offer a better chance to isolate NSCs and greatly facilitate the subsequent imaging steps.

### Larval brain dissection and mounting on the incubation chamber

**Timing: 20 min**

***Note:*** Culture medium must be pre-heated to 25°C prior to dissection and centrifuged at × *g* for 1 min to remove debris.

2. Larval brain dissection.
  a. Wash the larvae 3 times in PBS and transfer the larvae in 200 μL of culture media.
  b. Remove the brain and surrounding tissues under a binocular using fine tweezers.
  c. Separate the brain from the salivary glands and surrounding tissues.
3. Brain mounting in the incubation chamber.
  a. Gently aspirate the brain with a 200 Eppendorf pipet tip containing 50 μL of culture media and transfer the brain in the central well of the incubation chamber (Figure 1C).
  b. Immediately cover the brain with 200 μL of mineral oil in the incubation chamber.
  c. Remove excess culture medium by aspiration with a fine homemade glass capillary through the mineral oil layer (Figure 1D).

**CRITICAL:** Cut the end of the pipette tip off with a razor blade to avoid brain deformation during pipetting as the brain size may exceed the tip diameter.

***Note:*** Excess removal of culture media can trigger too much brain flattening on the coverslip and impair cell division. Aspiration is controlled under the binocular and should be stopped as soon as the brain lobes start to flatten.

***Note:*** Medium aspiration has the advantage of gently flattening the lobes and maintaining many NSCs in a close focal plane.

### Centrosome photo-ablation

4. NCS Imaging and centrosome photo-ablation.
  a. Image larval brain explants under an inverted Leica SP5 confocal microscope maintained at 25°C.
  b. Perform photo-ablation with a Mai-Tai two-photon infrared laser on a 2 × 2 μm region of interest at the centrosome in metaphase cells.

***Note:*** Use the following parameters for the multi-photon ablation; Trans: 80%, Gain: 100, offset: 36, iterations: 3 × 100 ms.

***Note:*** NSCs can be distinguished as being the largest spherical cells, with a diameter of 10–15 μm. They are positioned in the central part of the brain and located below the brain surface. We found that a plasma membrane marker such as PH-PLC-GFP is the best marker to accurately identify NSCs for non-experts.

**Figure 2:**
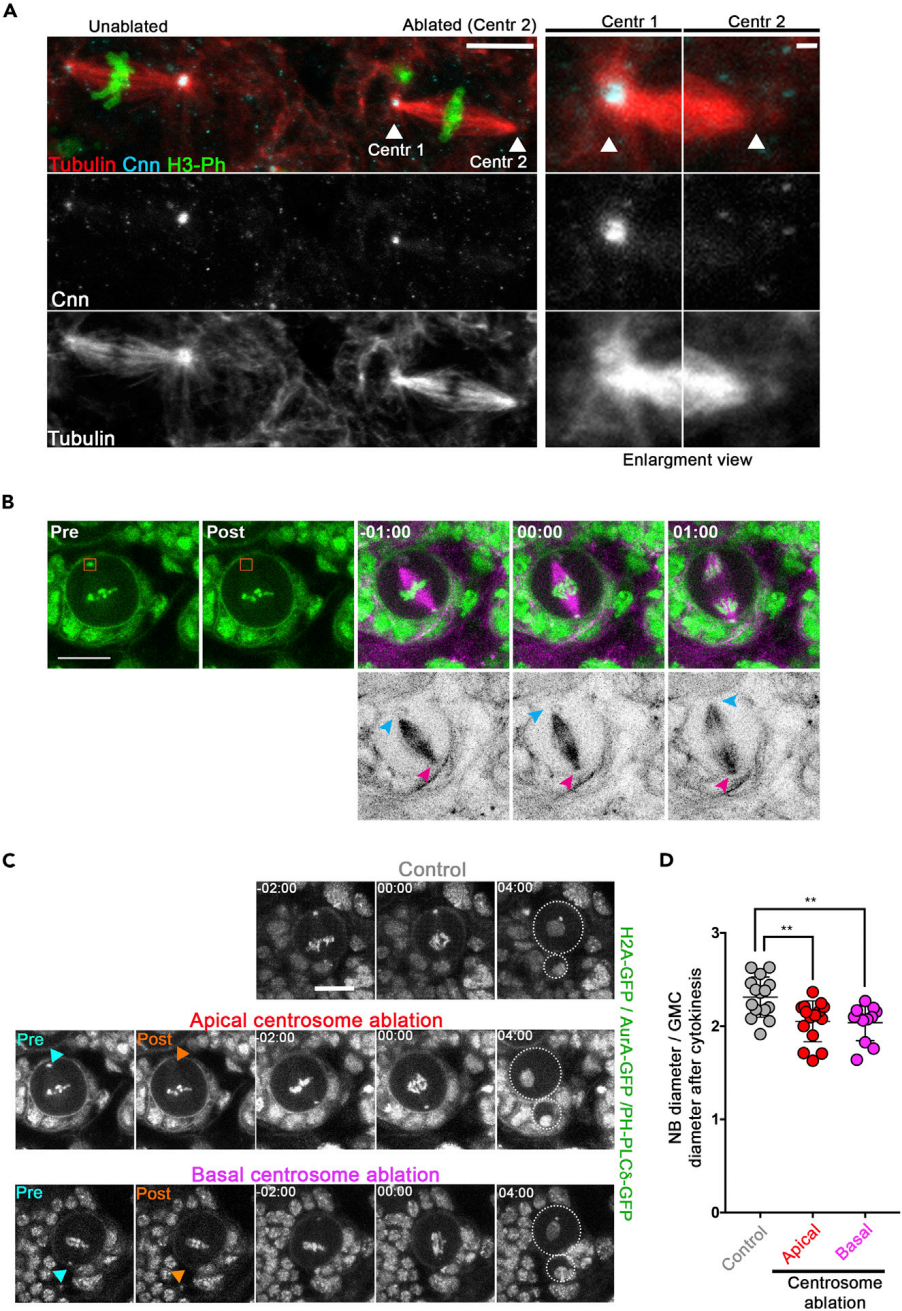
Laser ablation in NSCs (A) Control of centrosome ablation by immunostaining. The right panels show two metaphase NSCs from the same brain lobe. The centrosome of the right metaphase NSC was laser-ablated (right, Centr2) while the other was left intact (right, Centr1). Enlargement view of Centr1 and Centr2 regions corresponding to the “ablated” metaphase is also shown on the left. Tubulin is shown in red, centrosomin (Cnn) is shown in cyan and the phosphorylated Ser10 of Histone H3 (H3-Ph) is shown in green. Cnn and tubulin are also shown in monochrome in the middle and lower panels. Scale bars 5 μm (left panels) and 1 μm (right panels). (B) Dividing larval NSC expressing H2A-GFP, GFP-AurA, PH-PLCδ-GFP and RFP-α-tubulin subjected to centrosome ablation from metaphase to early anaphase. Pre and post-ablation images are shown in the green GFP channel to show disappearance of the centrosome labeled by GFP-AurA. The NSC is then imaged every 30 s to show RFP-α-tubulin and the GFP labeled proteins. Note that RFP-α-tubulin is also shown in monochrome in the lower panels. Scale bar: 10 μm. Time is min:s. The blue arrowhead shows the loss of the centrosome aster. The purple arrowhead shows the control non-ablated centrosome aster. (C) Cell division in a control NSC (top panels) and in NSCs in which the apical (middle panels, same cell shown in panel A) or the basal centrosomes (lower panels) were photo-ablated. The blue and orange arrowheads show the position of the centrosome before and after photo-ablation, respectively. GFP-AurA, H2A-GFP and PH-PLCδ-GFP are shown in monochrome. Scale bar: 10 μm. (D) Dot plot showing the mean (± s.d.) NSC diameter/GMC diameter ratio in controls (2.31 ± 0.22, *n=*15), in NSCs where the apical (2.05 ± 0.22, *n=*15) or the basal centrosomes (2.04 ± 0.19, *n=*12) were ablated. ∗∗: p<0.01 (Mann-Whitney test). Note that Figure 2B, C and D are reused data or panels from Thomas et al., 2021.

***Note:*** Centrosomes are identified by the presence of GFP-AurA dots located in the center of the RFP-α-tubulin microtubule aster during pro-metaphase and metaphase (Figure 2B).

***Note:*** Apical and basal NSC centrosomes can be differentiated by their respective size; the apical centrosome recruits more peri-centriolar components (e.g., GFP-AurA) and appears larger and brighter than the basal centrosome (Rebollo et al., 2007; Rusan and Peifer, 2007). In addition, the location of the GMCs that originate from previous NSC divisions (visible with the PH-PLCδ-GFP marker) enables to determine the position of the NSC basal side.

**CRITICAL:** To avoid tissue damage and photo bleaching, a maximum of 4 photo-ablation experiments are performed on different neuroblasts from the same brain.

### NSC imaging

5. Record NSC divisions with a 60 × 1.3 NA objective.

***Note:*** Acquire ten Z-series of 1 μm steps are acquired at 700 Hz (zoom 4) just before and after the photo-ablation at 30 s intervals. The image size acquired is 8 bits, 512 × 512 pixel, 2 frame-average (Figure 2C and Methods video S1).

Methods video S1. Asymmetric cell division in a control NSC or after basal centrosome ablation, related to Figure 2 and step 8 The left panel shows a control NCS. The right panel shows a NSC in which the basal centrosome was ablated. First and second images show pre- and post-ablation images respectively before acquisition. GFP-AurA, GFP-H2A and PH-PLCδ-GFP are displayed in gray. Scale bar: 10 μm. Time is min:s.

### Verification of centrosome ablation by immunostaining

6. Brains can be fixed immediately after photo-ablation to confirm centrosome depletion by immunostaining (Figure 2A).
  a. Gently aspirate the photo-ablated brain using a Pasteur pipette containing 50 μL of culture medium.
  b. Transfer the brain directly in 1 mL of fresh fixative solution (Bonaccorsi et al., 2011).
  c. Return the tube 3 times and incubate at 25°C for 20 min.
  d. Let the sample sink by capillarity and remove the fixative.
  e. Wash the fixed sample 3 times for 10 min on a rotating wheel in 1 mL of PBST.
  f. Process the brain for immunofluorescence using rabbit anti-Cnn (Zhang and Megraw, 2007), rat anti-tubulin and mouse anti-phosphorylated Histone H3 (Ser10) antibodies (Figure 2A) (Gallaud et al., 2014; Thomas et al., 2021).

### Image analysis

After recording, analyze NSCs divisions using the Fiji (ImageJ) software (Hiner et al., 2017; Rueden et al., 2017). The NCS and the GMC outskirts are visualized using the plasma membrane PH-PLCδ-GFP marker. The diameters of the newborn NSC and the GMC after cytokinesis are measured using the “line” tool on Fiji. The mean of at least two diameter measurements is performed for each cell. We use Fiji to make movies in controls and photo-ablated NSCs (Methods video S1).

7. Plot the data and perform appropriate statistical analyses using Prism (Figure 2D).
8. Select relevant time-lapse images to make figures and/or to export into videos (Figure 2 and Methods video S1).

## Expected outcomes

Peripheral astral microtubules (PAMs) represent a class of astral microtubules that are juxtaposed with the division furrow after anaphase. We found that inhibition of PAMs can be obtained using microtubule depolymerizing kinesins overexpression (Kinesins 8 and 13), loss of microtubule polymerizing proteins (Ensconsin/MAP7), or genetic removal of centrosomes (*sas-4*^*s2214*^ mutant). Destabilization of PAMs triggers the formation of an unstable asymmetric cleavage furrow that moves toward the apical pole, leading to less asymmetric daughter cells after cytokinesis. Removal of centrosome through photo-ablation also causes the depletion of astral microtubule populations (including PAMs). This impairs the position of the cleavage furrow, and ultimately modifies the daughter cell size asymmetry ratio. We found that the apical and basal centrosomes and their corresponding astral MT populations (PAMs) both contribute to asymmetric furrow positioning in *Drosophila melanogaster* NSCs (Figures 2C and 2D).

The technique we present here has the advantage of being very simple to implement and inexpensive. The *Drosophila* larval brain is a suitable model for imaging experiments: the tissue contains many proliferating NSCs located at the central brain surface and many probes are available to monitor fast cytoskeletal rearrangements. Moreover, the cell cycle is fast (45–60 min) and cell division occurs within 10 min. We therefore believe that other centrosome-dependent and independent processes can be monitored using our photo-ablation protocol during mitosis.

## Limitations

While we have successfully ablated centrosomes in metaphase cells, we found that ablation of centrosomes in prophase cells prevented anaphase onset. We have not tried to image photo-ablated NSCs for longer periods of time and further work would be needed to assess the long-term impact of centrosome laser photo-ablation on subsequent cell divisions.

## Troubleshooting

### Problem 1

Brain stressed during dissection: **NSCs do not divide.**

### Potential solution

The brain tissue is very sensitive to dissection. We found that the brain lobes should not be stretched or touched directly by the fine tweezers. Stressed NSCs are arrested in G2 or fail to exit mitosis. When a microtubule probe is used such as RFP-tubulin, bundles of microtubules are also frequently observed in addition to G2 or mitotic arrest.

### Problem 2

Brain stressed by excess flattening: **NSCs do not divide.**

### Potential solution

We recommend leaving a thin layer of culture media.

### Problem 3

Confirmation of centrosome photo-ablation.

Centrosomes are dynamic organelles that can move between the time its position was determined and the time the ablation occurs. It is also possible that the centrosome is bleached and not ablated. Control of successful centrosome ablation must then be performed. Samples are immediately fixed following ablation and processed for immunofluorescences using anti-centrosomin and anti-tubulin antibodies (Figure 2A) following a method described before (Gallaud et al., 2014; Métivier et al., 2020). The fixation method (see above) is time consuming and only one or two cells can be analyzed per brain. We found that the most straightforward method to state between centrosome photo bleaching (or failure to target a mobile centrosome) and centrosome photo-ablation was to monitor a dynamic centrosome component. Indeed, a past study has shown that fluorescence recovery after photobleaching for the centrosomal GFP-Aurora was fast (Full time recovery <10 s) indicating a quickly exchange between the cytoplasm the centrosome-associated GFP-AurA (Berdnik and Knoblich, 2002). Therefore, the disappearance of the GFP-AurA-labeled centrosome after more than 30 s post-ablation is a solid read-out of centrosome ablation.

### Problem 4

NSC destruction during photo-ablation.

### Potential solution

The metaphase centrosome is in a close proximity of the cell membrane during metaphase. The ROI for ablation needs to be carefully determined to avoid destruction of the NSC due to cytoplasmic membrane damage.

## Resource availability

### Lead contact

Further information and requests for resources and reagents should be directed to and will be fulfilled by the lead contact, [Régis Giet] (regis.giet@univ-rennes1.fr).

### Materials availability

This study did not generate new unique reagents.

## Data Availability

All data reported in this paper will be shared by the lead contact upon request. This paper study does not report original code.
